# Co‐Designing Lived Experience Guide Support for First Responder Mental Health: Defining the Role and Considerations for Implementation

**DOI:** 10.1111/hex.70461

**Published:** 2025-10-11

**Authors:** Hussain‐Abdulah Arjmand, Tim Peck, Meaghan Louise O'Donnell, Nicole Sadler, Anita Savic, Michelle Spinks, Michael Symons, Tracey Varker

**Affiliations:** ^1^ Phoenix Australia—Centre for Posttraumatic Mental Health Melbourne Australia; ^2^ Department of Psychiatry University of Melbourne Melbourne Australia

**Keywords:** co‐design, first responders, lived experience, mental health, treatment

## Abstract

**Background:**

Improving the mental health and well‐being of first responders has been a priority, leading to increased availability of treatments and services. However, access barriers continue to hinder the effectiveness of these services. Lived experience guides are mental healthcare service navigators who have real‐world experience working as a first responder and understand mental health challenges first responders experience. The aim of this study was to use an experience‐based co‐design methodology to define the lived experience guide role and identify important considerations for implementation in first responder settings.

**Methods:**

Nine current and former first responders from different agencies attended three co‐design workshops facilitated by the research team. First responders provided feedback and responses to specific question prompts. Responses were analysed within workshops and collaboratively grouped into emergent themes by first responders and facilitators.

**Results:**

Across workshops, responses were collated into six core themes: (i) the role and purpose of a lived experience guide; (ii) the importance of privacy, confidentiality and independence; (iii) appropriate selection and recruitment of guides; (iv) provision of training; (v) provision of resources and support; and (vi) evaluation.

**Conclusion:**

Lived experience guides represent a critical step towards better supporting first responders to overcome barriers and access appropriate services to improve mental health outcomes. This study provides useful insights for first responder agencies, government bodies and insurance providers focusing on first responder well‐being. Implementing the lived experience guide support stands to make a substantial impact on the mental health of first responders, contributing to more resilient and well‐supported emergency services personnel.

**Patient or Public Contribution:**

This study was co‐produced with a retired first responder with lived experience of mental health challenges who contributed substantially to study conceptualisation, methodology, investigation and manuscript preparation as a member of the author team. Additionally, current and former first responders with lived experience and knowledge of first responder mental health systems participated in co‐design workshops. Through these workshops, participants actively contributed to defining the lived experience guide role and identifying important considerations for implementation. Their involvement went beyond participation to collaborative interpretation of findings and discussion of implications.

**Acronyms**
AUD: Australian dollar
AV: Ambulance Victoria
CFA: Country Fire Authority
DEECA: Department of Energy, Environment and Climate Action
EBCD: experience‐based co‐design
FRV: Fire Rescue Victoria
PTSD: post‐traumatic stress disorder

## 1 Introduction

First responders (e.g., police, paramedics, firefighters and rescue workers) are regularly exposed to traumatic events due to the nature of their work, making them vulnerable to developing a mental health disorder [1]. Elevated rates of disorders such as depression, anxiety and post‐traumatic stress disorder (PTSD) have been reported in both current and former first responders [2, 3]. These rates are comparable to those observed in other workforces repeatedly exposed to traumatic events, such as military personnel and combat veterans [4], and are higher than those in the general civilian population [5].

Although internal (i.e., organisationally provided) and external mental health support services are available, first responders encounter numerous barriers to treatment and are often reluctant to seek help [6, 7]. Common barriers include lack of awareness or recognition of their own mental health symptoms, stigma associated with mental health difficulties and seeking help, poor service integration, and difficulties navigating complex treatment systems [8, 9]. In addition, first responders have high rates of dropout from mental health treatment [10], and first responders often do not think mental health services, programmes or treatments are useful or meet their needs [7, 11]. As such, there is a need to design and adapt supports and services to improve engagement with mental health treatment among first responders.

Lived experience guides, also referred to in previous research as ‘navigators’, offer a solution to access barriers encountered by first responders. This approach has been implemented in community settings to enhance treatment access and support populations with serious mental illness, resolve access barriers and connect with appropriate services [12, 13]. The role is characterised as a client‐centric partnership where the guide: (a) provides guidance around accessing treatment through complex healthcare systems; (b) facilitates timely access to treatment; and (c) fosters client self‐management through education, motivation and support [14, 15]. In previous research, guides (or navigators) have lived experience of seeking treatment for, and recovering from, a serious mental illness, which allows them to be uniquely empathetic to clients and fittingly experienced to facilitate hope, understanding and rapport [16, 17]. Previous research has shown that use of guides among specific populations with a serious mental illness (e.g., major depression, bipolar disorder, anxiety disorder, PTSD and schizophrenia) has been associated with increased mental healthcare access and utilisation, treatment adherence, and confidence in self‐management of healthcare [17, 18, 19]. As such, these models have the potential to be a cost‐effective way to improve access to mental health treatment among vulnerable populations [12].

From September 2023 to February 2024, the authorship team engaged first responder agencies through a series of consultation forums to better understand how to improve pathways to care for first responders. Agency members highlighted the importance of trust, noting that they are more likely to seek treatment when guided by someone who has first hand experience in their role. They emphasised the value of working with individuals who not only understand the unique culture of first responders but also possess knowledge of mental health symptoms and healthcare systems. As such, the consultation determined that there was a need to support first responders through lived experience guides and to define and understand the role in first responder settings through co‐design with members of the population (see Supplement A). To our knowledge, there have been no published reports on the development or implementation of lived experience guide support among first responders. As such, the aim of this study was to define the lived experience role in a first responder context and identify important considerations for implementation in this population. To achieve this, we used an experience‐based co‐design (EBCD) methodology to explore the perspectives, opinions and ideas of first responders with lived experience accessing mental health support for work‐related mental injury and first responders with knowledge of first responder mental healthcare systems. Across a series of co‐design workshops, we used collaborative and participatory methods to generate practical insights and recommendations from end users to help inform future policy, service development and prospective implementation efforts.

## 2 Methods

### 2.1 Core Team

This study was led by an experienced, retired first responder with lived experience of work‐related mental illness and the director of a mental health service for first responders (T.P.). This member leveraged strong networks across relevant first responder agencies and related organisations and coordinated all activities of this study. The lived experience member was accompanied by several supporting staff members, including three clinical specialists and one researcher. The clinical specialists led the design and facilitation of the workshop sessions, ensuring a comprehensive exploration of themes relevant to the implementation of lived experience guides in first responder settings. The researcher led the documentation of the co‐design process and activities, providing detailed records to facilitate reporting on the study outcomes and conclusion.

### 2.2 EBCD

To define the role of lived experience guides and identify important considerations for implementation in first responder contexts, an EBCD methodology was used. EBCD is a flexible method of designing health interventions, which has been increasingly used in healthcare settings [20, 21]. It is an adaptable approach which facilitates the understanding of problems and the generation of solutions through collaborative processes that engage key stakeholders. EBCD adopts a collective approach which puts prospective clients, service deliverers and relevant stakeholders at the heart of design, enabling them to collaborate and co‐design appropriate, feasible and needed interventions and services to improve outcomes [22]. Previous research has shown that engaging people with lived experience brings several benefits to service design and delivery [23, 24, 25]. When people with lived experience are engaged, the resulting work is more responsive to the needs and priorities of the target population, as well as more likely to be feasible, easily adopted, implemented and sustainable. In healthcare settings, the engagement of clients can also result in increased satisfaction with treatment and improved clinical outcomes [24].

### 2.3 Lived Experience Co‐Design Workshops

First responders with lived experience of accessing mental health treatment, or supporting others to access treatment, were identified via word of mouth, contacted by email or telephone, and invited to participate in the co‐design process. Three co‐design workshops were held, and attendees were financially compensated for travel costs, accommodation and meals. Attendees were also compensated $448 AUD per day for their time, paid via direct deposit, in line with established lived experience register rates [26]. Two workshops were held on consecutive days at the Victorian Emergency Management Institute in Melbourne, Australia, and a final workshop was held a month later at the same location. No work was conducted in the months between workshops.

All workshops followed established guidelines for engaging with lived and living experience members [27]. Workshops opened with discussions and reminders surrounding lived experience co‐design processes, the aims and purpose of the co‐design workshops, and creating an open space of honest and respectful communication between all involved [28]. As EBCD can be challenging in mental health contexts [22], workshops were designed to be sensitive and responsive to the needs of attendees, which was supported by the involvement of mental health professionals who facilitated the sessions and ensured a safe and supportive environment. Additionally, members were regularly reminded that there was no obligation to revisit or retell their personal stories of mental health challenges, but they were welcome to do so. The nature or extent of members' mental health was not screened, as they were determined not to affect their capacity to engage in the workshops. Workshops were co‐facilitated by the team leader and clinical experts of the core team.

The study prioritised engaging a diverse group of first responders with lived experience to collaboratively define the role of lived experience guides. The emphasis was placed on ensuring representation from key stakeholder groups rather than concentrating solely on numerical size. Attendees of the workshop were referred to as ‘lived experience co‐design members’. Members included two former police officers, two current serving police officers, one well‐being manager from Ambulance Victoria (AV), one representative from the Department of Energy, Environment and Climate Action (DEECA) who was also a former police officer, two retired members of Fire Rescue Victoria (FRV), and one representative from the volunteer‐based Country Fire Authority (CFA). All members maintained lived experience working as a first responder and experienced a work‐related mental injury for which they sought mental health treatment. One member did not have lived experience receiving mental health treatment; however, they had extensive experience working in first responder well‐being initiatives and supporting peers with work‐related mental health difficulties. One member was able to attend only the first workshop due to time commitments and was replaced by a colleague with a similar background to maintain continuity. Workshops were conducted from 9:30 AM to 3:30 PM, and participants were provided ample breaks throughout the day.

Co‐design workshops were structured around multiple topics. The first workshop commenced with a thought release activity, inviting attendees to form small groups and brainstorm any ideas related to their experiences seeking mental health support. Participants were asked to consider all aspects of current mental health supports available to first responders, identifying what worked well, what did not and opportunities for improvement. Written notes were then posted on whiteboards, categorised into similar groups, and discussed collectively. This activity served as a central reference for general ideas, allowing participants to revisit and draw upon these insights throughout subsequent topics. As new topics were discussed, members could refer back to the thoughts captured during the initial exercise to inform and guide their thinking on specific issues. Facilitators initiated and guided discussions on topics posing specific questions: (1) Why do we need lived experience guides? (2) What attributes should a lived experience guide possess? (3) How should we select lived experience guides? (4) What training should lived experience guides receive? (5) How should lived experience guides be supported? (6) What does success for a client look like? Each workshop day was tailored to cover specific topics, employing different approaches for idea generation and discussion to best suit the themes being explored.

Thematic synthesis of member feedback and responses was informed by principles of participatory action research and collaborative data analysis [29, 30]. This approach centred around collaborative exploration within workshops where members actively contributed to the generation and refinement of themes in real time with each other and workshop facilitators. Members shared thoughts and ideas through individual and group written responses (e.g., using butcher paper or adhesive notes), small group discussions, and presenting summaries to workshop attendees. Written responses were pinned and displayed on whiteboards for group reflection, and similar ideas, thoughts and responses were collaboratively analysed and discussed, and grouped into emergent themes by co‐design members and facilitators. All themes and outcomes identified in workshops were carefully documented by an experienced member of the research team.

## 3 Results

**Figure 1:**
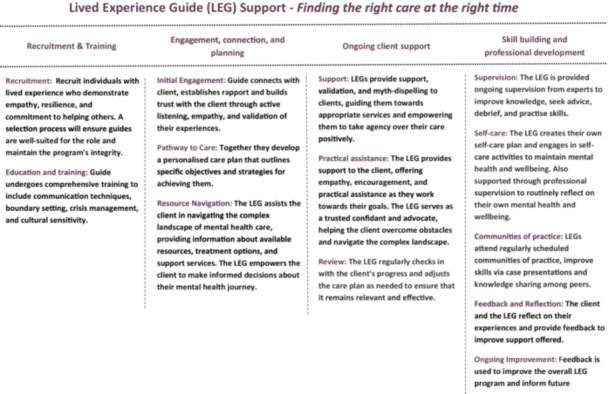
Lived Experience Guide (LEG) Support—Finding the right care at the right time.

Across workshops, all members provided rich input and contributed to the aim of the study. Core themes identified within workshops included (1) the role and purpose of a lived experience guide; (2) privacy, confidentiality and independence; (3) selection and recruitment of lived experience guides; (4) training; (5) resources and support; and (6) evaluation of the lived experience guide support. During the workshop, topics and ideas discussed were organised into a preliminary ‘journey map’ to collectively aggregate members' input; the map illustrates the roles and expected experiences lived experience guides across the lifespan of the support provided (see Figure 1).

### 3.1 The Role and Purpose of a Lived Experience Guide

Members highlighted perceived gaps in service consolidation among the array of existing services. Although multiple organisational and external services are available to first responders, they can be perceived as fragmented and vary in degrees of confidence in effectiveness, confidentiality, visibility and ease of access. This can lead to confusion and an overwhelming amount of information for first responders to parse. As such, a primary role of lived experience guides should be to reduce this burden by streamlining information about supports and services and highlighting relevant options available to first responders.

Members emphasised that the role should address first responders' reluctance to seek mental health support. Accordingly, the role should help reduce stigma, dispel common myths about treatment and its effectiveness, validate client experiences, and motivate individuals to access appropriate care. They highlighted that lived experience guides should offer hope and options, bridge gaps in service access, and empower first responders to take an active role in managing their mental health with appropriate guidance and support.

Lived experience guides should represent a ‘soft entry point’ into seeking mental health assistance and should be ‘light touch’ in nature. They should offer flexibility in supporting clients, either in person or online (e.g., videoconferencing), at appropriate intervals suited to client needs (e.g., weekly). It must be clear to clients that providing mental health treatment is not within the scope of a lived experience guide's role; rather, they are there to support and offer direction towards the best referral options. Members highlighted that matching clients with lived experience guides of similar age, gender and occupational background can help facilitate rapport; however, services should also be responsive to clients who may have specific requests. Finally, members explained that the lived experience guide position should be a paid role in line with current lived experience workforce frameworks [31].

### 3.2 Privacy, Confidentiality and Independence

Members described privacy and confidentiality as central requirements of a lived experience guide support. They strongly endorsed the idea of guides operating from an organisation independent of first responder agencies. Members noted that many first responders fear ‘grapevine communication’, or gossip, in the workplace, which precipitates reluctance to approach a colleague or peer supports to express mental health challenges and seek help. There is worry that information will circulate among staff and senior management, which could threaten career progression, workplace identity and perceived capacity for duty. Members noted that this can be a significant barrier for first responders, which an independent organisation could overcome. Members suggested that a proportion of first responders would be more comfortable engaging an externally based lived experience guide who would be easier to engage privately and involve fewer boundary conflicts as compared to internal organisational services.

Members also identified that confidentiality and security regarding personal data and information capture are crucial to sustain client trust. Members noted that a key task of lived experience guides will be recording session notes. This may capture sensitive and personal information regarding names, addresses and key contacts. It is essential that session notes are stored appropriately, using secure and accessible means such as online digital platforms with high levels of security and data protection. Handwritten notes, or notes saved locally on a computer, were deemed as unsecure and would deter first responder clients who are highly protective of their privacy and information.

### 3.3 Selection and Recruitment of Lived Experience Guides

Former first responders were highlighted as ideal lived experience guide candidates compared to current‐serving members who maintain close involvement within first responder organisations. Notwithstanding, no strict demographic characteristics were identified by members that would exclude individuals with experience working as a first responder (e.g., rank, years of service, current or ex‐serving). Experiencing a prior mental health condition was not deemed to be an essential requirement for the role; however, it was acknowledged that this could promote rapport, connection and understanding between the client and guide.

Members noted that organisations employing lived experience guides should have recruitment processes that include careful assessment of candidates' mental health status to ensure stability and safety. Members suggested that recruitment should involve psychometric testing of mental health symptoms, face‐to‐face interviews with mental health clinicians, and referee checks. Psychometric testing enables a reliable and valid assessment of mental health symptom experience, and clinical evaluation by mental health clinicians can assess suitability and inform whether symptoms are likely to impact performance. In line with this, members described desirable and undesirable personal characteristics which should be considered during face‐to‐face interviews to mitigate risks and minimise poor outcomes. These characteristics are outlined in Table 1.

**Table 1:** Desirable and undesirable personal characteristics for recruitment of lived experience guides derived from collaborative group discussions during co‐design workshops.

Desirable	Undesirable
Capacity to manage personal journey (if impacted by trauma)	Maintaining negative feelings towards previously employing agency
Self‐awareness of personal capacity and ability to manage over‐commitment and burnout	Harbouring bitterness against workers' compensation insurance agencies
Ability to maintain professionalism through discussion of trauma	A reputation for gossip or destructive behaviours
Knowledge of first responder organisations and culture	Living with unresolved personal trauma or mental health issues
Strong, authentic interpersonal communication skills	
Strong self‐management skills	
Flexible with time	

### 3.4 Resources and Support

To ensure optimal support is provided by lived experience guides, members discussed the resources and ongoing support that guides would need to perform their role effectively. Members outlined the need for a co‐ordinator to manage and oversee administrative elements of the role (e.g., recruitment, allocation and employment contracts) and provide day‐to‐day line management of workload and activities tasked to guides. Concurrently, lived experience guides should be supported with regular supervision or reflective practice sessions with a supervisor where they can debrief and seek expert advice on aspects directly relating to their support role. Supervision also functions to protect and maintain the mental health of guides who may be impacted through the support they provide. In line with this, members discussed the importance of providing avenues for guides to take a break or opt out of providing support for a short while if required. Members with prior experiences providing peer support highlighted the ill effects that having a limited ability to reduce their support load or stop seeing clients for a while when needed can have. Implementing such breaks can be facilitated through joint discussions with the co‐ordinator and supervisor. Members also discussed that a lived experience guides should be supported through communities of practice, where guides can discuss case presentations, share information and knowledge, connect with peers, and feel part of a cohesive workforce.

Important resources for lived experience guides included clear documentation of processes and governance structures. Such documentation should outline for guides who they directly report to, where they can seek advice or assistance, what to do in emergency situations, and details of key contacts. Documentation should also include ‘cheat sheet’ like resources which guides can utilise to improve the quality of support provided; this may include documentation outlining first responder organisational structures, workers' compensation processes, lists of available mental health services within agencies, and lists of externally available services. There should also be well‐defined sessional note‐taking and reporting processes, data security measures, and adherence to safe working procedures. Comprehensive documentation protocols were highlighted as essential elements to support lived experience guides, who will require secure means of storing client information, reporting on key outcomes of interest, and maintaining session notes. Guides must maintain detailed session notes for each individual interaction. Session notes serve multiple purposes, including documentation of the session content, progress tracking for clients, and providing a record of interactions to protect against legal issues. Having comprehensive session notes not only ensures accountability and professionalism but also provides a reference point for guides to revisit if any challenging situations arise during their sessions. It is imperative that these notes be recorded on a secure online database to protect client data.

### 3.5 Training

Members strongly advocated for the provision of comprehensive and supportive training for lived experience guides. They emphasised the importance of a well‐structured training programme where the format is tailored to first responder preferences. There was consensus that face‐to‐face training would be most favoured as it allows for direct interaction and experiential learning. In‐person training should include a blend of theory and practice, incorporating small group discussions, role‐plays, scenarios and real‐life experiences based on authentic situations to enhance learning and skill application. The use of case studies was recommended to deepen understanding of issues and individual stories and to resemble approaches used in first responder training. Training facilitators should comprise experienced mental health clinicians who have worked with first responder populations and are skilled in working with trauma‐related mental health issues. Members also agreed that face‐to‐face sessions could be preceded by online training, such as trauma‐informed care, which provides foundational knowledge that complements in‐person training. Further, members highlighted that training should also be ongoing, and communities of practice can function as a medium for continued skill development, as well as running periodic booster training sessions to maintain, refine and further develop skills.

With regard to training content, members emphasised that training should be grounded in the latest research and informed by recognised frameworks (e.g., trauma‐informed practice, lived experience guidelines and peer support guidelines). Several key topics were identified as essential to cover, which are outlined in Table 2. Overall, members advocated for a holistic training approach that equips guides with the necessary knowledge and skills to provide effective support for first responder populations.

**Table 2:** Essential content for training lived experience guides identified through group attendee feedback and group discussions.

Essential training for lived experience guides
Defining roles, responsibilities and boundaries of lived experience guides
Ethical and effective provision of support via personal lived experience
Effective communication skills (e.g., active and empathetic listening)
Psychoeducation and knowledge of common mental health presentations in first responders
Understanding mental healthcare systems and referral pathways available to first responders
Low‐impact mental health interventions (e.g., grounding, breathing exercises, circuit‐breakers and helpful thinking strategies)
Identification, assessment and referral of clients' risk of harm to self or others
Self‐care and reflective practices

### 3.6 Evaluating Lived Experience Guide Support

Members highlighted that the impacts of lived experience guide supports should be measurable to enable evaluation of effectiveness. Discussions surrounding evaluation focused on defining successful outcomes. Various metrics were identified, emphasising that success can look different for different individuals. Members noted that first responders will vary in their willingness and capacity to engage with mental health services and that success will manifest differently across this spectrum. For example, two first responders with similar symptom profiles and concerns around privacy and confidentiality may seek support from a lived experience guide; these clients, however, may have different levels of willingness and capacity to access treatment. A first responder with high willingness and capacity may only require a single meeting with a guide to understand their treatment options, which may lead to quick access to treatment. In contrast, a first responder with low willingness and capacity may require ongoing support with a guide who can, over time, dispel myths, provide motivation, and offer different referral pathways. This client may not access treatment immediately; however, they may continue meeting with their guide and seek more information, and their willingness to access treatment may improve. Such outcomes were considered successful as they move first responders closer to accessing treatment. As such, several key metrics were distilled, which are outlined in Table 3.

**Table 3:** Metrics of successful outcomes of lived experience guide support identified through attendee feedback and collaborative group discussion.

Key metrics of success
Number of clients who successfully access mental health treatment
Number of clients who begin seeking mental health treatment
Change in the levels of willingness of clients to engage in mental health treatment services
Number of sessions with a lived experience guide

## 4 Discussion

There has recently been increased focus on the availability of mental health treatments and services for first responders [32, 33, 34]. However, efforts to provide services have been fettered by numerous access barriers [6]. Lived experience guides represent a potential solution to overcome such gaps. Unfortunately, there is a dearth of published reports on the initiation or development of lived experience guide supports specifically for first responders. Accordingly, the aim of this study was to define the lived experience role in a first responder context and identify important considerations for implementation in this population. Using EBCD methods, several core themes were identified across a series of workshops, including a description of the role and purpose of a lived experience guide; the importance of privacy, confidentiality and independence; appropriate selection and recruitment of guides; provision of resources and support; provision of training; and evaluation.

Members of the co‐design team affirmed the need for lived experience guides. Many first responders do not engage in treatment services when experiencing mental health issues [7, 35]. Reasons for this include a lack of awareness of services, while members of the co‐design team highlighted that an abundance of options can be overwhelming [6, 8, 35]. This can lead to uncertainty about what each service involves and difficulty determining which is most suitable. Further, willingness to engage in treatments is often hindered due to mental health stigma, myths about impacts on employment, concerns about insurance schemes, as well as worries that treatments will not be effective, efforts will be a waste of time, or they do not deserve treatment [3, 8, 36]. A primary purpose of lived experience guides should therefore be to enhance awareness of available services, organise and simplify information, direct clients towards options suited to their needs, and provide guidance around complex systems processes. Concurrently, guides should also promote willingness to seek and access treatments by providing hope through sharing stories, motivation and clarity around all aspects of help‐seeking from a lived experience point of view.

Previous implementations of service guides have employed individuals with lived experience of seeking treatment and recovering from mental illness [17, 18]. Although experience of a mental illness was not deemed in this study to be an essential requirement of lived experience guides, it was highlighted as a useful component of lived experience, which could foster a connection between the client and the guide. In such cases, it is important to consider how declaring lived experience of a mental health condition can present challenges. In contrast, an essential requirement was having lived experience working as a first responder, as it signals trust, cultural competence and understanding of occupational experience, which are critical to developing rapport in this population [36, 37]. However, members of the co‐design team highlighted that lived experience could also lead to unfavourable outcomes if guides are not appropriately screened before recruitment. Lived experience working as a first responder can foster undesirable qualities for the role, such as distrust or bitterness towards one's agency, which may reduce impartiality and negatively bias support provided [8]. Further, high prevalence rates of mental conditions in first responders mean that lived experience guides may live with trauma or untreated mental injuries resulting from their work, which co‐design team members speculated could aggravate client symptoms if not managed appropriately [2, 3]. It is important to note, however, that addressing these concerns during recruitment requires careful balance: employers cannot discriminate on the basis of disability, including mental health conditions, yet they also carry a duty of care to both clients and lived experience guides. Concerns may be balanced through thorough and structured recruitment, training and supervision frameworks (e.g., role‐specific interviews, reflective exercises and ongoing professional development). Such approaches can focus on recruiting based on role fitness (via in‐depth interviews) and demonstration of relevant experience or skills such as active listening, boundary setting, recovery orientation and self‐awareness, rather than a focus on mental health histories. Previous research in mental health settings has not commented on concerns surrounding recruitment and selection processes [16, 17, 38]; however, it has been noted that an interview process has been used in previous research to assess guides' skills in fostering recovery, self‐determination, and listening and problem‐solving skills [18].

Consistent with prior research, members emphasised privacy, confidentiality and independence as key requirements of lived experience guide support [6, 36]. This differs from community settings where these concerns are less pronounced [13, 39]. Although similar in function, members highlighted that a distinguishing feature between lived experience guides and organisational supports (e.g., peer supports) should be independence from clients' agency. First responders can be apprehensive about disclosing sensitive information to colleagues and have expressed concern over perceived professional ramifications [7, 8]. Seeking support within one's own workplace can also present boundary conflicts. Members articulated that first responders may feel like an inconvenience for taking up colleagues' time, requesting assistance or burdening them with personal problems. As a result, they may avoid engaging peer supporters or may not disclose fully during sessions, which may explain the limited use and effectiveness reported in previous first responder research [7, 40]. Knowing that lived experience guides maintain a paid, independent, dedicated role of support may help reduce the above‐mentioned concerns among reluctant first responders and encourage support‐seeking behaviours. It is important to note, however, that the purpose of lived experience guides is not to replace internally offered services, but to provide additional choice and an independent option for first responders who may be especially hesitant to seek help.

Previous implementations of service guides have included training and resources to support their roles, including tailored resource guides, practice manuals and training workshops [16, 17, 18, 41]. In prior documentation of service guides in the literature, details provided on training delivered to service guides are often insufficient to be replicated [42]. High‐level descriptions of training content vary across studies, but topics covered often include scope of practice, cultural awareness, interviewing skills, insurance systems, case assessment and client management [15, 41, 43]. The skills required by service guides to undertake their role described in previous research include goals‐based planning, problem‐solving skills, relapse management, harm reduction, crisis management and trauma‐informed care [17], which aligns with the training content overview developed in the current study. Similarly, the training delivery methods identified in the current study align with prior research, which has highlighted the utility of role‐playing, modelling, coaching and feedback to optimise learning and fidelity for service guides [17].

Members noted that supervision is an impactful resource for lived experience guides. Supervision is common practice among lived experience workforces and is considered an integral part of optimal professional practice in health practitioners [44, 45]. Supervision can develop theoretical knowledge and practical skills, reduce psychological burden, improve satisfaction and job retention, and promote adherence to ethical standards and best practices [44, 46, 47]. Additionally, supervision can facilitate reflective practice, allowing guides to critically evaluate their work and continuously improve skills [48].

Finally, characterising success is important for evaluating the impacts of lived experience guides. Evaluating mental health interventions ensures they are effective, and resources are utilised efficiently. Previous studies of navigation services have reported on metrics which align with the key outcomes identified in this study. These include outcomes such as service utilisation rates, the number of treatment sessions or appointments attended, time taken to access services, and self‐management attitudes and behaviours [43, 49, 50]. Members of the co‐design team articulated broad conceptualisations of success, highlighting that success can vary across a spectrum of willingness and capacity to engage with treatment services. Therefore, in addition to the above‐mentioned utilisation metrics, future evaluations should consider quantification of treatment‐seeking actions or behaviours, as well as measures of readiness or willingness to engage in treatments. Establishing standardised and operational definitions for these outcomes will be a crucial task for future research. Evaluation is vital to provide stakeholders and prospective funders with outcomes which exhibit meaningful impact. This is important for expanding programmes and broadening the reach of effects imparted by lived experience guides.

## 5 Conclusion

Lived experience guides and service navigator roles have been implemented in mental healthcare settings among individuals with serious mental illness (e.g., schizophrenia, bipolar disorder, major depression and anxiety disorders) with positive effects, helping individuals navigate complex care systems to more easily access appropriate treatment or support. Our EBCD research defines the lived experience role in a first responder context and outlines considerations for implementation in this population. The insights gained from our study may offer valuable guidance for first responder agencies, government bodies and insurance providers focused on occupational health and safety, injury prevention and management, and workers' compensation. Implementing lived experience guides supports could serve as an effective strategy to overcome access barriers, reduce stigma and enhance mental health outcomes among first responders. Lived experience guides may have the potential to improve the well‐being of first responders by providing tailored support that acknowledges their unique experiences and challenges. Piloting this form of support and evaluating its effectiveness is essential, and this represents a promising future direction of research. Such pilots could explore specific metrics, such as willingness to access treatment, treatment‐seeking behaviours, and engagement with mental health treatment. These pilots could provide preliminary evidence of the effectiveness of lived experience guides and inform best practices for broader implementation.

## Author Contributions

**Hussain‐Abdulah Arjmand:** conceptualisation, methodology, investigation, writing – original draft, writing – review and editing. **Tracey Varker:** conceptualisation, writing – original draft, writing – review and editing. **Meaghan Louise O'Donnell:** conceptualisation, writing – original draft, writing – review and editing. **Tim Peck:** conceptualisation, methodology, investigation, project administration. **Nicole Sadler:** conceptualisation, writing – review and editing. **Michael Symons:** investigation, methodology, writing – review and editing. **Anita Savic:** investigation, methodology, writing – review and editing. **Michael Symons:** investigation, methodology, writing – review and editing.

## Disclosure

The views expressed in this report do not necessarily represent the views of the Department of Health or the Ministers of Health.

## Ethics Statement

This study employed a co‐design approach guided by principles of participatory action research and collaborative qualitative data analysis. Participants were engaged as co‐design members in workshops where they provided thoughts and ideas through written responses on non‐permanent media (i.e., a whiteboard), small group discussions and group reflections. The workshop was classified as a community engagement activity rather than formal research; therefore, ethics approval was not sought. No identifiable data were collected or retained.

## Consent

Formal (written) consent was not obtained, as the workshop was conducted as a community engagement activity rather than formal research. However, all participants were informed of the purpose and nature of the activity and provided verbal consent to participate. Attendance and active involvement were taken as an indication of informed, voluntary participation.

## Conflicts of Interest

The authors declare no conflicts of interest.

## Supporting information

Supporting material.

## Data Availability

All data were generated, discussed and analysed during the co‐design workshop through non‐permanent media (e.g., whiteboards and sticky notes), small group discussions and group reflections. As part of the co‐design approach, no data were retained or stored beyond the workshop itself. All relevant insights are reported within the manuscript.
