# Supplementary material for: Co‐Designing Lived Experience Guide Support for First Responder Mental Health: Defining the Role and Considerations for Implementation
Source: Health Expect. 2025 Oct 11;28(5):e70461. doi: 10.1111/hex.70461 (PMC12514453; doi:10.1111/hex.70461)
Supplement: Supplementary file 1 — Supporting material. [file HEX-28-e70461-s001.docx]

**Supplement A**

**Engagement with agency-level stakeholders**

A series of forums was held with stakeholders from multiple first responder agencies including Victoria Police, Ambulance Victoria, Forest Firefighters Victoria, State Emergency Service, Fire Rescue Victoria, and the Emergency Services Telecommunications Authority, as well as various insurance agency representatives and other affiliated organisations. The first forum brought together stakeholders to generate ideas on how to improve referral pathways to mental health treatment so that first responders can access needed treatments. Members of the core team facilitated this workshop and engaged stakeholders through discussion. Participants were broken up into smaller groups to identify solutions and share with other groups. Four separate solution concepts were produced: (1) improve first responder awareness and literacy of mental health services available, (2) establish clarity about the range and purpose of services (e.g., prevention, early intervention, or treatment), (3) incorporate lived experience storytelling to motivate first responders to access treatment, and (4) improve relationships and connections between service providers. The forum collectively acknowledged the need for action and explored a range of preliminary ideas and solutions to resolve this challenge.

A second forum was held with stakeholders from the initial forum to advance the ideas and solutions generated in the first forum. The second forum also included a panel of three former first responders with lived experience of seeking support for mental health issues and accessing treatment who shared their journeys and discussed the challenges they faced. With active involvement of the lived experience panel and agency stakeholders, a first responder persona was developed that articulated the motivation behind seeking treatment, common barriers to treatment, and experiences accessing available resources and services. With consideration of the solution concepts generated in the first forum, stakeholders were separated into groups and directed to formulate an intervention, service idea, or plan of action that could be implemented to improve access and engagement with treatment services among first responders. Similar service ideas were produced across groups (e.g., “lived experience champions” and “cross-organisational buddy”), which focused on provision of independent referral advice and service navigation support by individuals with (1) lived experience working as a first responder and (2) lived experience accessing mental health support for work-related mental injuries. Together, core themes of these ideas were combined into a single concept for a lived experience guide support service.

A third forum was conducted to review and refine the idea with stakeholders to discuss next steps in developing the concept. Consensus was reached that concept development should be informed by co-design with first responders who possess relevant knowledge of first responder mental health care systems or lived experience seeking mental health treatment through such systems. Stakeholders highlighted that co-design members should include a combination of current and ex-serving first responders, volunteer and paid first responders, a diverse range of ages and gender, first responders familiar with pathways to accessing support, and stakeholders from first responder organisations.
